# Different regulation of limb development by p63 transcript variants

**DOI:** 10.1371/journal.pone.0174122

**Published:** 2017-03-23

**Authors:** Manabu Kawata, Yuki Taniguchi, Daisuke Mori, Fumiko Yano, Shinsuke Ohba, Ung-il Chung, Tomomi Shimogori, Alea A. Mills, Sakae Tanaka, Taku Saito

**Affiliations:** 1 Sensory & Motor System Medicine, Faculty of Medicine, The University of Tokyo. 7-3-1 Hongo, Bunkyo-ku, Tokyo, Japan; 2 Bone and Cartilage Regenerative Medicine, Faculty of Medicine, The University of Tokyo. 7-3-1 Hongo, Bunkyo-ku, Tokyo, Japan; 3 Center for Disease Biology and Integrative Medicine, Faculty of Medicine, The University of Tokyo. 7-3-1 Hongo, Bunkyo-ku, Tokyo, Japan; 4 Shimogori Research Unit, RIKEN Brain Science Institute. 2–1 Hirosawa, Wako, Saitama, Japan; 5 Cold Spring Harbor Laboratory. Cold Spring Harbor, New York, United States of America; Texas A&M University, UNITED STATES

## Abstract

The apical ectodermal ridge (AER), located at the distal end of each limb bud, is a key signaling center which controls outgrowth and patterning of the proximal-distal axis of the limb through secretion of various molecules. Fibroblast growth factors (FGFs), particularly Fgf8 and Fgf4, are representative molecules produced by AER cells, and essential to maintain the AER and cell proliferation in the underlying mesenchyme, meanwhile Jag2-Notch pathway negatively regulates the AER and limb development. p63, a transcription factor of the p53 family, is expressed in the AER and indispensable for limb formation. However, the underlying mechanisms and specific roles of p63 variants are unknown. Here, we quantified the expression of p63 variants in mouse limbs from embryonic day (E) 10.5 to E12.5, and found that *ΔNp63γ* was strongly expressed in limbs at all stages, while *TAp63γ* expression was rapidly increased in the later stages. Fluorescence-activated cell sorting analysis of limb bud cells from reporter mouse embryos at E11.5 revealed that all variants were abundantly expressed in AER cells, and their expression was very low in mesenchymal cells. We then generated AER-specific p63 knockout mice by mating mice with a null and a flox allele of *p63*, and *Msx2-Cre* mice (*Msx2-Cre;p63*^*Δ/fl*^). *Msx2-Cre;p63*^*Δ/fl*^ neonates showed limb malformation that was more obvious in distal elements. Expression of various AER-related genes was decreased in *Msx2-Cre;p63*^*Δ/fl*^ limb buds and embryoid bodies formed by p63-knockdown induced pluripotent stem cells. Promoter analyses and chromatin immunoprecipitation assays demonstrated Fgf8 and Fgf4 as transcriptional targets of ΔNp63γ, and Jag2 as that of TAp63γ. Furthermore, TAp63γ overexpression exacerbated the phenotype of *Msx2-Cre;p63*^*Δ/fl*^ mice. These data indicate that ΔNp63 and TAp63 control limb development through transcriptional regulation of different target molecules with different roles in the AER. Our findings contribute to further understanding of the molecular network of limb development.

## Introduction

The first structure in vertebrate limb formation is the limb bud, swellings in the lateral body wall of mouse embryos at embryonic day (E) 9.5 [1]. The apical ectodermal ridge (AER) is located at the distal end of each limb bud and consists of ectodermal cells [2]. The AER is a key signaling center which controls outgrowth and patterning of the proximal-distal axis of the limb through secretion of various molecules [2].

Fibroblast growth factors (FGFs), such as Fgf4, Fgf8, Fgf9, and Fgf17, are representative molecules specifically produced by AER cells. They are essential to maintain the AER and cell proliferation in the underlying mesenchyme [3–5]. Their roles in the AER are partially redundant, but double knockout of Fgf8 and Fgf4 results in malformation of the distal limb elements [6]. Jag2, a canonical Notch ligand, is also expressed in the AER [7, 8]. Disruption of Jag2 or Notch signaling causes hyperplasia of the AER due to a decrease in programmed cell death and consequential impairment of limb development [7, 8], indicating that the Jag2-Notch pathway negatively regulates the AER and limb growth. In addition, R-spondin2 (Rspo2), a secreted protein that activates Wnt/β-catenin signaling, is expressed in the AER, and its knockout leads to impaired limb growth [9, 10]. In terms of transcriptional regulation, distal-less homeobox (Dlx) 5 and Dlx6 are expressed in the AER, and the double mutant mice display limb malformation with decreased expression of Fgf8 [11]. Msh homeobox (Msx) 1 and Msx2 are expressed in and around the AER, which are also involved in limb development [12, 13]. Dlx5 and Dlx6 are thought to be upstream to Msx1 and Msx2 in the AER, and they control a signaling network that regulates limb outgrowth and patterning [14].

p63, a transcription factor of the p53 family, is a potent regulator of cell proliferation, survival, and apoptosis in various cell types and tissues [15]. Two major transcript variants are TAp63 with an N-terminal transactivation (TA) domain and ΔNp63 without this domain [16]. Although the ΔNp63 form was previously believed to be dominant negative, it has become clear that it has transcriptional activities [17, 18]. Both TAp63 and ΔNp63 can be alternatively spliced at the 3′ terminus to produce α-, β-, and γ-variants [19].

p63 is essential for normal formation of the epidermis, and p63-deficient mice are born with shiny, transparent skin and die within several hours, possibly because of dehydration [20, 21]. Another feature of this mouse is marked limb defects. In p63-deficient neonates, their forelimbs are truncated and the hindlimbs are almost absent [20, 21]. In the forelimbs of the mutants, all distal components (autopods) are absent, whereas mid components (zeugopods) are heterogeneously defective and proximal components (stylopods) are hypoplastic. Notably, p63 knockout results in a severely hypoplastic AER and consequential impairment of limb bud formation [20, 21]. In humans, mutations in the p63 gene cause several kinds of diseases with limb deformities, including split-hand/split-foot malformation 4 (SHFM4), ectrodactyly and ectodermal dysplasia, and cleft lip/palate syndrome 3 (EEC3), and ankyloblepharon-ectodermal defects-clefting (AEC) syndrome [19].

p63 is highly expressed in the AER, and its deletion impairs AER formation [20, 21]. Previous studies show that Dlx5 and Dlx6 are transcriptional target genes of p63 during limb development [22], and Fgf8 and Msx1 are thought to be downstream of p63 because their expression is diminished by p63 knockout [20, 21]. However, the molecular mechanisms underlying the regulation of limb development by p63 and the specific roles of its transcript variants are still unknown.

Here, we quantified expression of p63 transcript variants in the AER and limb mesenchyme through fluorescence-activated cell sorting (FACS) using respective tissue-specific Cre mice and Cre-dependent reporter mice. We further examined the *in vivo* roles of p63 using p63-flox mice and inducible p63 transgenic mice, as well as transcriptional regulation of AER-related genes by p63.

## Materials and methods

### Ethics statement

All experiments using mice were performed according to the protocols approved by the Animal Care and Use Committee of The University of Tokyo (approval number; M-P12-131). Cervical dislocation was used as a euthanasia method. All efforts were made to minimize suffering.

### Mice

Mice were maintained on a C57BL/6J background and bred in an environmentally controlled specific pathogen free room at 23 ± 2°C with 50–60% relative humidity under a 12-hour light/dark cycle. Noon of the day on which a vaginal plug was seen was considered as E0.5.

We obtained *Msx2-Cre* mice [23] from the Mutant Mouse Resource Research Center (Davis, CA, USA), *Prrx1-Cre* [24] and *Rosa-CAG-LSL-tdTomato* (*Ai14*) mice [25] from the Jackson Laboratory (Bar Harbor, ME, USA), and *CAG-Cre* mice [26] from RIKEN BRC (Ibaraki, Japan). The mice with *p63* floxed allele were generated as described previously [27]. To generate *CAG-EGFP-TAp63γ* mice, a transgene was constructed as described previously [28]. DNA purification and microinjection were performed according to standard protocols. Genotyping was performed by PCR using genomic DNA from mouse tails, KOD FX DNA polymerase (Toyobo, Osaka, Japan), and specific primers (S1 Table).

### Real-time RT-qPCR

Total RNA was isolated with an RNeasy Mini Kit (Qiagen, Hilden, Germany) and 1 μg was reverse transcribed using ReverTra Ace qPCR RT Master Mix with gDNA Remover (Toyobo). Real-time RT-qPCR was performed with a Thermal Cycler Dice Real Time System Single (Takara, Otsu, Japan). Each PCR contained 1× THUNDERBIRD SYBR qPCR Mix (Toyobo), 0.3 mM specific primers, and 20 ng cDNA. The mRNA copy number of each specific gene in the total RNA was calculated using a standard curve generated by serially diluted plasmids containing PCR amplicons. The copy number was normalized to rodent total RNA (Thermo Fisher Scientific, Waltham, MA, USA) with mouse *β-actin* as the internal control. All reactions were run in triplicate. The primer sequences are shown in S2 Table.

### FACS analysis of mouse limb bud cells

Forelimb buds of *Prrx1-Cre;Ai14* E11.5 embryos were dissociated into single cells using 0.1% trypsin (Sigma-Aldrich, St Louis, MO, USA) and 0.1% collagenase type II (Sigma-Aldrich) at 37°C for 30 minutes in a humidified atmosphere with 5% CO_2_. The dissociated cells were sorted into positive or negative populations using the 561 nm (yellow-green) laser of a BD FACSAria Fusion cell sorter (BD Biosciences, Franklin Lakes, NJ, USA). Total RNA were purified with an RNeasy Micro Kit (Qiagen).

### Alizarin red and alcian blue staining

Skin, viscera, and adipose tissue of neonatal mice were removed and fixed in 100% ethanol for 4 days. The samples were incubated at 37°C for 2 days with 0.015% alcian blue 8GS (Sigma-Aldrich), and then with 0.002% alizarin red S (Sigma-Aldrich) and 1% KOH in the dark for 12 hours. Samples were cleared in a 1% KOH:glycerol series (20:80 and 50:50) until the soft tissues were dissolved. The specimens were stored in 80% glycerol.

### WISH analysis

WISH was performed according to standard protocols [29]. The following probes were used: 881 bp *p63* probe (nucleotides 875 to 1,755, NCBI Reference Sequence: NM_001127261.1), 1,034 bp *Jag2* probe (nucleotides 592 to 1,625, NM_010588.2), and 473 bp *Msx2* probe (nucleotides 94 to 566, NM_013601.2). The nucleotide sequence of the probe for *Fgf8* [30] was generously provided by Prof. Hiroki Kurihara (The University of Tokyo, Tokyo, Japan). Digoxigenin (DIG)-labelled riboprobes for these genes were generated by a DIG RNA labelling kit (SP6/T7) (Roche, Basel, Switzerland).

### Establishment and maintenance of *p63*^*fl/fl*^ iPS cells

Fibroblasts were isolated from *p63*^*fl/fl*^ mouse embryos (E12.5) according to standard protocols [31] and applied to iPS cell generation using concentrated vesicular stomatitis virus-G-retroviral supernatant as described previously [32, 33]. *p63*^*fl/fl*^ iPS cells were cultured on mitomycin C-inactivated *p63*^*fl/fl*^ mouse embryonic fibroblasts as feeder cells at 37°C in a humidified atmosphere with 5% CO_2_ in ES medium [StemSure D-MEM (High Glucose) with Phenol Red and Sodium Pyruvate (Wako, Osaka, Japan), 15% StemSure Serum Replacement (Wako), 2 mM L-glutamine (Thermo Fisher Scientific), 1% (vol/vol) nonessential amino acids (Thermo Fisher Scientific), 0.1 mM 2-mercaptoethanol (Sigma-Aldrich), 50 U/mL penicillin (Sigma-Aldrich), 50 mg/mL streptomycin (Sigma-Aldrich), and 1,000 U/mL leukaemia inhibitory factor (Wako)].

### Formation of embryoid bodies

Colonies of mouse iPS cells were gently detached with a 0.1× Trypsin/EDTA solution (Sigma-Aldrich) and cultured in suspension on Petri dishes for 5 days in ES medium with 100 nM all-trans retinoic acid (Cayman, Ann Arbor, MI, USA).

### Construction of expression vectors

Coding sequences of *ΔNp63γ* and *TAp63γ* were amplified from mouse cDNA and cloned into pCMV-3Tag-1a vector (Agilent Technologies, Santa Clara, CA, USA). DNA sequencing was performed to verify each construct.

### Culture of B16 melanoma cells

B16 melanoma cells were purchased from RIKEN BRC and cultured at 37°C in a humidified atmosphere with 5% CO_2_ in RPMI 1640 medium (Thermo Fisher Scientific) with 10% (vol/vol) fetal bovine serum (Sigma-Aldrich).

### Luciferase assays

Fragments of *Fgf8* (from –3,750 to +51 bp relative to the TSS, *Jag2* (from +423 to +2,309 bp), and *Fgf4* (from –3,468 to +0 bp) genes were amplified from mouse genomic DNA and cloned into pGL4.10[luc2] vector (Promega, Madison, WI, USA). The short fragments of *Fgf8* (–3,650 to –2,631, –1,238 to –839, –839 to –469, and –469 to –201 bp), *Jag2* (+423 to +604, +582 to +1,242, +1,223 to +2,042, and +2,023 to +2,282 bp), and *Fgf4* (–3,460 to –3,030, –2,887 to –2,475, –1,430 to –920, and –570 to –170 bp) were cloned into pGL4.10[luc2] vector containing a miniP. Mutant constructs lacking p63 consensus motifs were constructed by PCR. B16 melanoma cells were cultured in 48-well plates and cotransfected with 130 ng/well reporter vectors, 65 ng/well effector vectors, and 2 ng/well pRL-TK (Promega) as an internal control using Lipofectamine 2000 Transfection Reagent (Thermo Fisher Scientific). After 48 hours, luciferase activities were detected by the Dual-Luciferase Reporter Assay System (Promega). All data are shown as the ratio of firefly luciferase activity to Renilla luciferase activity. All assays were performed in triplicate.

### ChIP assays

Mouse ES cells under feeder-free conditions [34, 35] were transfected with 3×FLAG-tagged *ΔNp63γ* or *TAp63γ* expression vectors. After 72 hours, the cells were fixed in 1% formaldehyde for 10 minutes, and ChIP was performed using 5×10^7^ cells from each sample, according to a previously published protocol [36]. We used 25 μg of a monoclonal antibody that recognizes FLAG (Clone M2; Sigma-Aldrich) for each assay.

qPCR was performed with THUNDERBIRD SYBR qPCR Mix. Fold enrichment was calculated by normalizing the ChIP sample against the input, and the target region against the control region as follows [37]. ΔCt = Ct (ChIP)–Ct (input); ΔΔCt = ΔCt (target region)– ΔCt (control region); fold enrichment = 2^−ΔΔCt^. All reactions were run in triplicate. The primers used for ChIP-qPCR are described in S3 Table. The negative control primers were designed in the upstream region of *Aldh1a2* (S3 Table).

### Statistical analyses

The unpaired two-tailed Student's t test was used to assess the statistical significance of experimental data. *P*-values of less than 0.05 were considered significant.

## Results

### Expression of p63 variants during limb development

**Fig 1:**
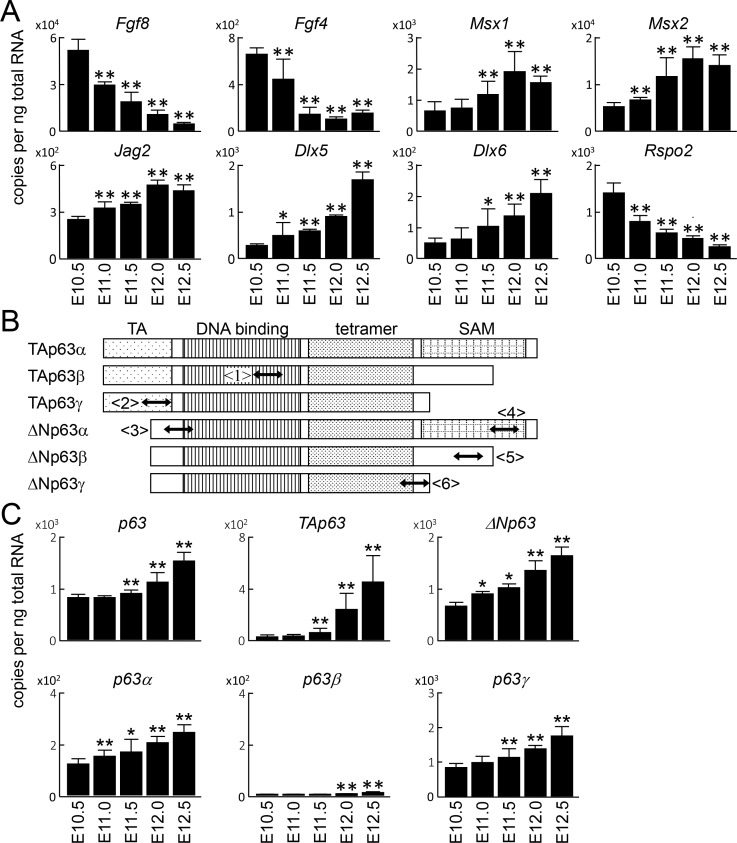
Expression of p63 variants during limb development. (A) mRNA levels of apical ectodermal ridge (AER)-related genes in whole limbs during development. Error bars indicate s.d. (*n* = 3 biological replicates). *P < 0.05, **P < 0.01 vs. E10.5 (unpaired two-tailed Student's t test). (B) Schematic representation of p63 transcript variants. Two-way arrows indicate amplicons of RT-qPCR detecting all forms <1>, TA form <2>, ΔN form <3>, α form <4>, β form <5>, and γ form <6>. (C) mRNA levels of *p63* and its transcript variants in whole limbs during development. Error bars indicate s.d. (*n* = 3 biological replicates). *P < 0.05, **P < 0.01 vs. E10.5 (unpaired two-tailed Student's t test).

Initially, we examined expression of p63 transcript variants in limb growth. We first harvested whole forelimbs of wild-type (WT) mouse embryos at E10.5, E11.0, E11.5, E12.0 and E12.5, and analyzed the mRNA levels of AER-related genes (Fig 1A). Expression of *Fgf8*, the most AER-specific gene among these markers, was decreased and accompanied by the normal decline in the relative size of the AER as the limb develops (Fig 1A). *Fgf4* and *Rspo2* expression was also decreased in the time course, while *Msx1*, *Msx2*, *Jag2*, *Dlx5*, and *Dlx6* expression was increased (Fig 1A). To examine the expression of p63 transcript variants in these samples, we designed primer sets common for all variants or specific for each variant (Fig 1B). Expression of *p63* was increased gradually as development progressed (Fig 1C). For N-terminal variants, *ΔNp63* was more abundantly expressed at all stages, while *TAp63* was increased by more than 10-fold from E10.5 to E12.5 (Fig 1C). Among the three C-terminal variants, *p63γ* was most abundant at all stages, and *p63α* was expressed at about one-seventh of *p63γ* expression (Fig 1C). *p63β* was scarcely detected (Fig 1C).

### Expression of p63 variants in the AER and limb mesenchyme

**Fig 2:**
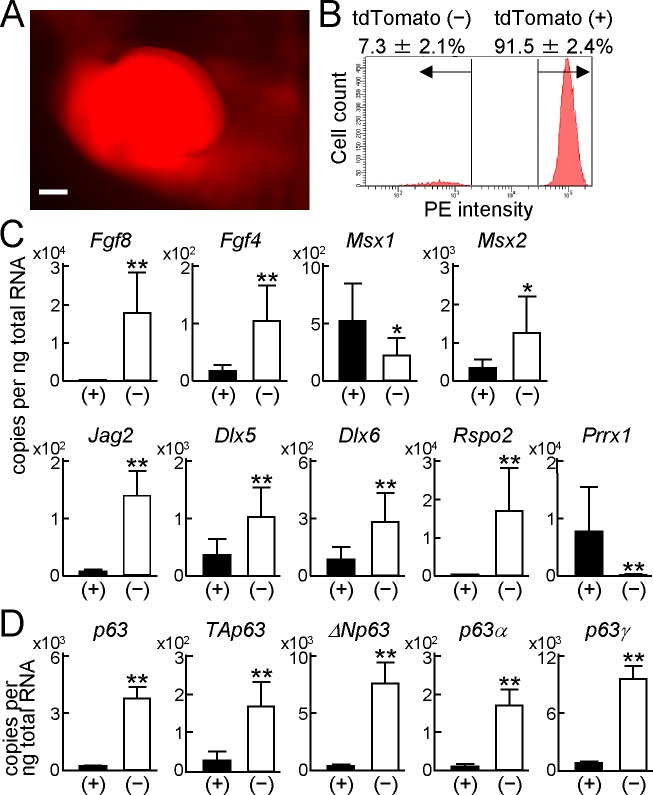
Expression of p63 variants in the AER and limb mesenchyme. (A) Fluorescence image of a forelimb bud in a *Prrx1-Cre;Rosa-CAG-LSL-tdTomato* (*Ai14*) E11.5 embryo. Scale bar, 200 μm. (B) Flow cytometric analyses of forelimb bud cells from *Prrx1-Cre;Ai14* E11.5 embryos. Error bars indicate s.d. (*n* = 3 biological replicates). (C) mRNA levels of AER-related genes in tdTomato positive (+) or negative (−) cells from the forelimb buds of *Prrx1-Cre;Ai14* E11.5 embryos. Error bars indicate s.d. (*n* = 3 biological replicates). *P < 0.05, **P < 0.01 vs. (+) (unpaired two-tailed Student's t test). (D) mRNA levels of *p63* and its transcript variants in tdTomato positive (+) or negative (−) cells from the forelimb buds of *Prrx1-Cre;Ai14* E11.5 embryos. Error bars indicate s.d. (*n* = 3 biological replicates). **P < 0.01 vs. (+) (unpaired two-tailed Student's t test).

To identify p63 expression in the AER and limb mesenchyme, we used *Prrx1-Cre* mice, which express Cre in the mesenchyme of embryonic limb buds [24], and Cre reporter mice *Rosa-CAG-LSL-tdTomato* (*Ai14*) [25]. In the limb bud of *Prrx1-Cre;Ai14* E11.5 embryos, fluorescence was intensely observed in the limb mesenchyme (Fig 2A). We next harvested forelimb buds from these embryos, dissociated them into single cells by trypsin and collagenase, and performed FACS analysis. The cells were divided into two groups: a major group with strong fluorescence [tdTomato (+)] and a minor group with weak fluorescence [tdTomato (−)] (Fig 2B). When we collected these cells and examined the expression of marker genes, *Fgf8*, *Fgf4*, *Msx2*, *Jag2*, *Dlx5*, *Dlx6*, and *Rspo2* were strongly expressed in tdTomato (−) cells, while *Msx1* and *Prrx1* were expressed in tdTomato (+) cells (Fig 2C). *p63* expression in the tdTomato (−) cells was about 20 times higher than that in tdTomato (+) cells, and all transcript variants were expressed more abundantly in the tdTomato (−) cells (Fig 2D). Among the variants, *ΔNp63* and *p63γ* were the abundant N- and C-terminal variants in the tdTomato (−) cells, respectively (Fig 2D). All these data using *Prrx1-Cre;Ai14* mice indicated appropriate sorting of AER and mesenchymal cells, and more abundant expression of *ΔNp63* and *p63γ* in the AER cells.

### Impaired development of limbs in AER-specific p63 knockout mice

Because all transcript variants were strongly expressed in AER cells and their expression in mesenchymal cells was very low, we deleted p63 in the AER using the mice homozygous for *p63* floxed alleles (*p63*^*fl/fl*^), which are fully wild-type in function [27]. First, we generated p63 heterozygous knockout mice (*p63*^*Δ/+*^) by mating *CAG-Cre* mice [26] with *p63*^*fl/fl*^ mice and removing the *CAG-Cre* allele by crossing with WT mice. Similar to p63-deficient neonates [20, 21], limb formation was markedly impaired in *p63*^*Δ/Δ*^ neonates (S1A and S1B Fig). Homozygous and heterozygous knockout of p63 were confirmed by RT-qPCR (S1C Fig).

**Fig 3:**
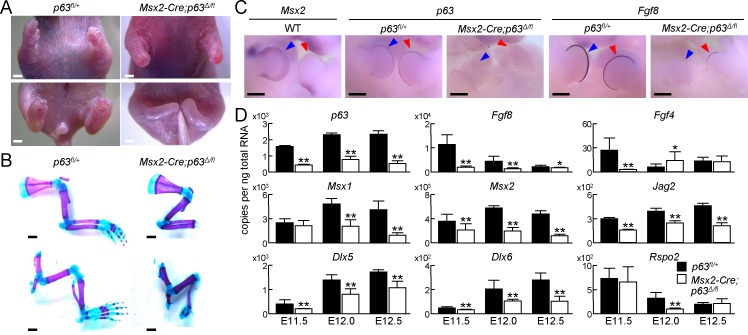
Impaired development of distal limbs in AER-specific p63 knockout mice. (A) Gross appearances of upper extremities (top) and lower extremities (bottom) of *p63*^*fl/+*^ and *Msx2-Cre;p63*^*Δ/fl*^ neonates. Scale bar, 1 mm. Images are representative of *n* = 3 mice per genotype. (B) Double staining with alizarin red and alcian blue of upper extremities (top) and lower extremities (bottom) of *p63*^*fl/+*^ and *Msx2-Cre;p63*^*Δ/fl*^ neonates. Scale bar, 1 mm. Images are representative of *n* = 3 mice per genotype. (C) Whole-mount in situ hybridization (WISH) of *Msx2* in a WT E11.5 embryo, and *p63* and *Fgf8* in *p63*^*fl/+*^ or *Msx2-Cre;p63*^*Δ/fl*^ E11.5 embryos. Blue and red arrowheads indicate forelimb and hindlimb buds, respectively. Scale bar, 500 μm. Images are representative of *n* = 3 embryos per condition. (D) mRNA levels of *p63* and AER-related genes in whole limbs obtained from *p63*^*fl/+*^ or *Msx2-Cre;p63*^*Δ/fl*^ embryos at E11.5, E12.0 and E12.5. Error bars indicate s.d. (*n* = 3 biological replicates). *P < 0.05, **P < 0.01 vs. *p63*^*fl/+*^ (unpaired two-tailed Student's t test).

We next employed *Msx2-Cre* mice, another AER-specific Cre mouse strain [23]. *Msx2-Cre;p63*^*Δ/fl*^ embryos displayed hypoplasia of autopod and distal zeugopod (Fig 3A and 3B), but the impairment of limb growth in *Msx2-Cre;p63*^*Δ/fl*^ neonates was milder than that in *p63*^*Δ/Δ*^ neonates (Fig 3A and 3B, S1A and S1B Fig). Whole-mount *in situ* hybridization (WISH) using a WT embryo showed that *Msx2* expression was weak around the tip of the AER, and expression of *p63* and *Fgf8* was slightly detected around the same area in *Msx2-Cre;p63*^*Δ/fl*^ embryos (Fig 3C), although it was completely diminished in p63 null embryos [20, 21]. To quantify mRNA levels, we harvested whole limbs from *p63*^*fl/+*^ and *Msx2-Cre;p63*^*Δ/fl*^ littermate embryos at E11.5, E12.0 and E12.5. *p63* expression was decreased to one-third to one-fifth by *Msx2-Cre* mediated knockout, and the AER-related genes were downregulated significantly (Fig 3D).

### Down-regulation of AER-related genes by p63 deletion

**Fig 4:**
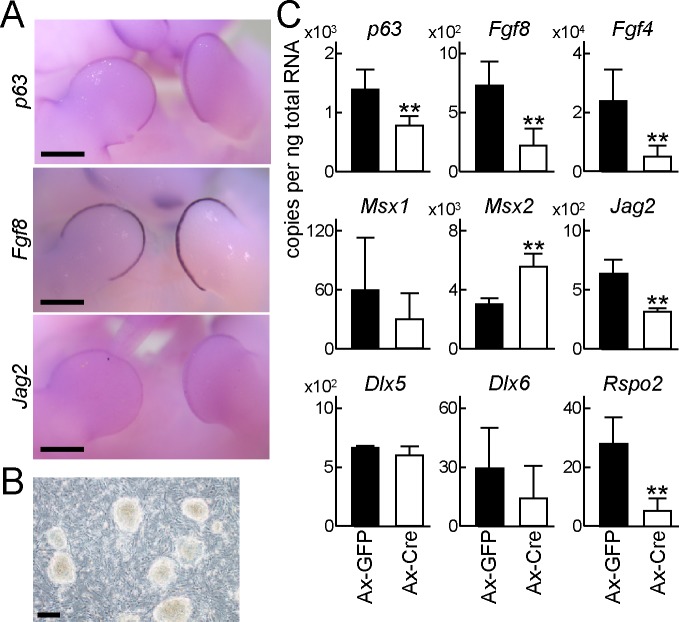
Down-regulation of AER-related genes by p63 deletion. (A) WISH of *p63*, *Fgf8*, and *Jag2* in a WT E11.5 embryo. Scale bar, 500 μm. Images are representative of *n* = 3 embryos per condition. (B) Induced pluripotent stem (iPS) cells generated from *p63*^*fl/fl*^ embryonic fibroblasts by retroviral introduction of Klf4, Oct4, Sox2 and Myc. Scale bar, 100 μm. (C) mRNA levels of *p63* and AER-related genes in embryoid bodies formed by *p63*^*fl/fl*^ iPS cells infected with green fluorescent protein (GFP) (Ax-GFP) or Cre (Ax-Cre) adenoviruses. Error bars indicate s.d. (*n* = 3 biological replicates). **P < 0.01 vs. Ax-GFP (unpaired two-tailed Student's t test).

During limb organogenesis, Fgf8 is the most essential factor in the AER [6]. Fgf8 knockout, particularly combined with Fgf4 knockout, results in severe impairment of limb formation [6]. Fgf8 is thought to be a downstream molecule of p63, because its expression is markedly diminished in p63 null limb buds [20]. However, it is still unknown whether Fgf8 is a direct transcriptional target of p63. Furthermore, Jag2, a representative Notch ligand, is expressed in the AER [8], and its deletion causes hyperplasia of the AER [7], in contrast to Fgf8 knockout. It has been previously reported that Jag2 is a downstream gene of p63 in thymic development [16], but the relationship between these genes has not been revealed in the AER. Tissue-specific deletion of p63 diminished the expression of *Fgf8* and *Jag2* (Fig 3D), and WISH showed co-expression of *p63*, *Fgf8*, and *Jag2* (Fig 4A), indicating that both may be target genes of p63. Because Fgf8 and Jag2 have opposite effects in limb organogenesis, we further analyzed transcriptional regulation of both genes by p63.

To perform loss-of-function analysis of p63 *in vitro*, we generated induced pluripotent (iPS) cells from *p63*^*fl/fl*^ mouse embryonic fibroblasts (Fig 4B). Embryoid bodies were formed at 1 day after adenoviral transduction of green fluorescent protein (GFP) or Cre recombinase, and RNA was collected from whole embryoid bodies after an additional 5 days in culture. Among the AER-related genes, expression of *Fgf8* and *Jag2* was significantly downregulated by Cre introduction (Fig 4C).

### Transcriptional regulation of Fgf8 and Fgf4 by ΔNp63γ

**Fig 5:**
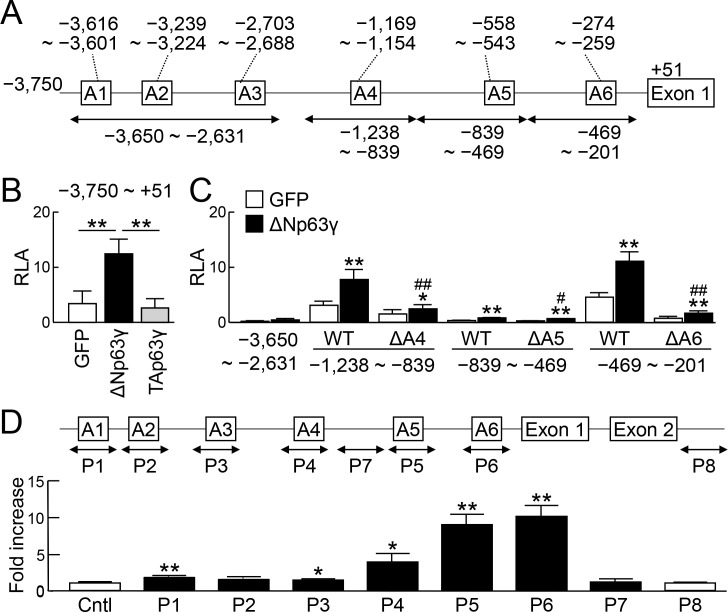
Transcriptional regulation of Fgf8 by ΔNp63γ. (A) 5′-end flanking region up to −3,750 bp from the transcription start site (TSS) of the mouse Fgf8 gene. Six consensus sequences for p63 binding in this region are shown as A1−6. (B) Luciferase activities in B16 melanoma cells co-transfected with a luciferase reporter gene construct containing a fragment (−3,750 bp to +51 bp) of the Fgf8 gene and an expression vector for GFP, ΔNp63γ, or TAp63γ. RLA, relative luciferase activity. Error bars indicate s.d. (*n* = 3 biological replicates). **P < 0.01 (unpaired two-tailed Student's t test). (C) Luciferase activities in B16 melanoma cells co-transfected with luciferase reporter gene constructs containing the indicated fragments ligated to a minimal promoter (miniP) and an expression vector for GFP or ΔNp63γ. Error bars indicate s.d. (*n* = 3 biological replicates). *P < 0.05, **P < 0.01 vs. GFP. ^#^*P* < 0.05, ^##^*P* < 0.01 vs. WT with ΔNp63γ (unpaired two-tailed Student's t test). (D) ChIP-qPCR using lysates of mouse ES cells transfected with 3×FLAG-tagged ΔNp63γ. The amplicon of each primer set is indicated as P1–8 in the scheme. Negative control primers were designed in the upstream region of *Aldh1a2* (Cntl). Error bars indicate s.d. (*n* = 3 biological replicates). *P < 0.05, **P < 0.01 vs. Cntl (unpaired two-tailed Student's t test).

We further examined the molecular mechanisms underlying the Fgf8 induction by p63. We found six p63 consensus motifs (A1–6) in the proximal 5′-end flanking region of the transcription start site (TSS) of the mouse Fgf8 gene (Fig 5A). When we cloned the 3.8 kb region into a luciferase reporter vector and performed a luciferase assay, the promoter activity was strongly increased by ΔNp63γ (Fig 5B). We then amplified four fragments of −3,650 to −2,631, −1,238 to −839, −839 to −469, and −469 to −201 bp including A1−3, A4, A5 and A6, respectively (Fig 5A), and cloned them into luciferase reporter vectors with a minimal promoter (miniP). The enhancer activity was significantly increased by ΔNp63γ in the reporter vector with −1,238 to −839, −839 to −469, or −469 to −201 bp (Fig 5C). Furthermore, the activity was diminished by deletion of the p63 consensus motif in each region (Fig 5C). To examine binding of p63 protein to these regions, we transfected an expression vector for 3×FLAG-tagged ΔNp63γ into mouse embryonic stem (ES) cells under feeder-free conditions, and performed a chromatin immunoprecipitation (ChIP) assay using an anti-FLAG antibody. We designed six primer sets, P1–6, spanning A1–6, respectively, and two primer sets, P7 and 8, which did not span the p63 consensus motifs (Fig 5D). qPCR showed high enrichment in P6 and P5, and low enrichment in P4 (Fig 5D).

We next performed a similar investigation of Fgf4. It is known to compensate for the role of Fgf8 in limb development [38], and its expression was significantly decreased in the limb buds of AER-specific p63 knockout embryos (Fig 3D) and embryoid bodies formed by p63-knockdown iPS cells (Fig 4C). We found four p63 consensus motifs (B1–4) in the proximal 5′-end flanking region of the TSS of the mouse Fgf4 gene (S2A Fig). Similar to Fgf8, when we cloned the 3.5 kb region into a luciferase reporter vector and a performed luciferase assay, the promoter activity was strongly increased by ΔNp63γ (S2B Fig). We then amplified four fragments of −3,460 to −3,030, −2,887 to −2,475, −1,430 to −920, and −570 to −170 bp, which included each motif (S2A Fig), and cloned them into luciferase reporter vectors with a miniP. The enhancer activity was significantly increased by ΔNp63γ in all reporter vectors (S2C Fig). Among them, the activity was significantly decreased by deletion of the p63 consensus motif in B1, B2 and B4 (S2C Fig).

### Transcriptional regulation of Jag2 by TAp63γ

**Fig 6:**
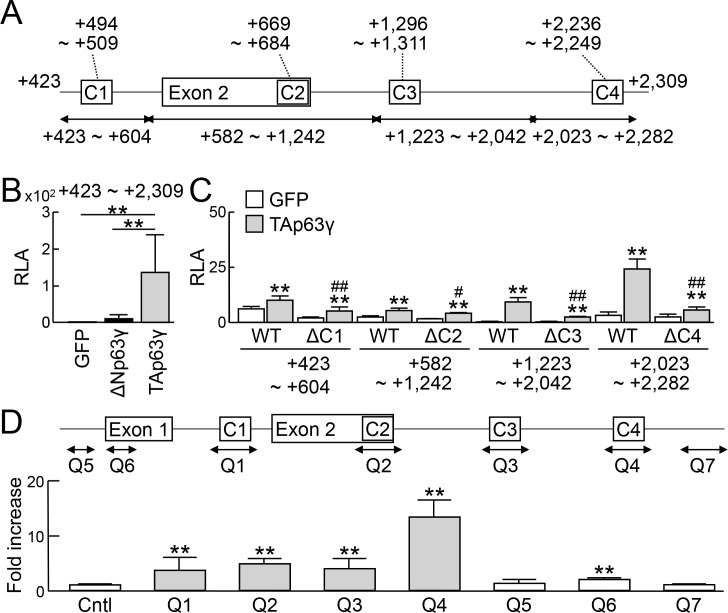
Transcriptional regulation of Jag2 by TAp63γ. (A) Region around exon 2 of the mouse Jag2 gene. Four consensus sequences for p63 binding in this region are shown as C1–4. (B) Luciferase activities in B16 melanoma cells co-transfected with a luciferase reporter gene construct containing a fragment (+423 bp to +2,309 bp) of the Jag2 gene ligated to a miniP and an expression vector for GFP, ΔNp63γ, or TAp63γ. RLA, relative luciferase activity. Error bars indicate s.d. (*n* = 3 biological replicates). **P < 0.01 (unpaired two-tailed Student's t test). (C) Luciferase activities in B16 melanoma cells co-transfected with luciferase reporter gene constructs containing the indicated fragments ligated to a miniP and an expression vector for GFP or TAp63γ. Error bars indicate s.d. (*n* = 3 biological replicates). *P < 0.05, **P < 0.01 vs. GFP. ^#^*P* < 0.05, ^##^*P* < 0.01 vs. WT with TAp63γ (unpaired two-tailed Student's t test). (D) ChIP-qPCR using lysates of mouse ES cells transfected with 3×FLAG-tagged TAp63γ. An amplicon of each primer set is indicated as Q1–7 in the scheme. The negative control primers were designed in the upstream region of *Aldh1a2* (Cntl). Error bars indicate s.d. (*n* = 3 biological replicates). *P < 0.05, **P < 0.01 vs. Cntl (unpaired two-tailed Student's t test).

We next examined the mechanisms of Jag2 induction by p63. Although there is no p63 consensus motif in the proximal 5′-end flanking region of the TSS, we found four motifs (C1–4) in and around exon 2 (Fig 6A). Notably, in contrast to the transactivation of Fgf8 and Fgf4 promoters by ΔNp63γ, the enhancer activity of the reporter vector containing the region of +423 to +2,309 bp with the miniP was markedly enhanced by TAp63γ (Fig 6B). All four reporter vectors showed significant transactivation by TAp63γ, and deletion of the respective consensus motif decreased the activity (Fig 6C). A ChIP assay also displayed significant enrichment by the primer sets containing C1-4 (Fig 6D). Among them, the region containing C4 showed the strongest activation in the luciferase assay, and the highest enrichment in the ChIP assay (Fig 6C and 6D).

### Exacerbation of limb formation in AER-specific p63 knockout mice by TAp63γ overexpression

**Fig 7:**
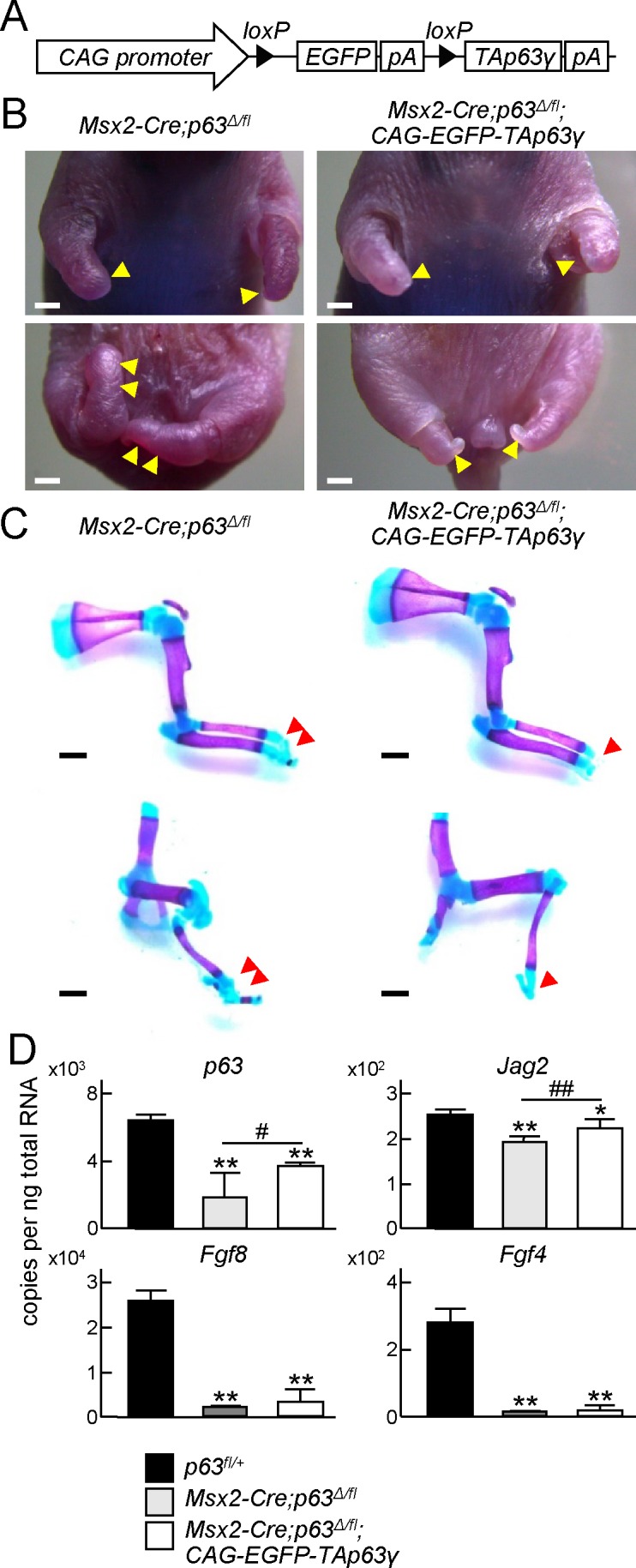
Exacerbation of limb formation in AER-specific p63 knockout mice by TAp63γ overexpression. (A) Transgene construct of *CAG-EGFP-TAp63γ*. The transgenic mouse expressed TAp63γ in a Cre recombinase-dependent manner. pA, polyA. (B) Gross appearances of upper extremities (top) and lower extremities (bottom) of *Msx2-Cre;p63*^*Δ/fl*^ and *Msx2-Cre;p63*^*Δ/fl*^;*CAG-EGFP-TAp63γ* neonates. Yellow arrowheads indicate hypoplastic autopods. Scale bar, 1 mm. Images are representative of *n* = 3 mice per genotype. (C) Double staining with alizarin red and alcian blue of upper extremities (top) and lower extremities (bottom) of *Msx2-Cre;p63*^*Δ/fl*^ and *Msx2-Cre;p63*^*Δ/fl*^;*CAG-EGFP-TAp63γ* neonates. Red arrowheads indicate hypoplastic autopods. Scale bar, 1 mm. Images are representative of *n* = 3 mice per genotype. (D) mRNA levels of *p63*, *Fgf8*, *Fgf4* and *Jag2* in whole limbs obtained from *p63*^*fl/+*^, *Msx2-Cre;p63*^*Δ/fl*^, and *Msx2-Cre;p63*^*Δ/fl*^;*CAG-EGFP-TAp63γ* embryos at E11.5. Error bars indicate s.d. (*n* = 2 biological replicates). *P < 0.05, **P < 0.01 vs. *p63*^*fl/+*^. ^#^*P* < 0.05, ^##^*P* < 0.01 vs. *Msx2-Cre;p63*^*Δ/fl*^ (unpaired two-tailed Student's t test).

Considering our data, p63 may regulate AER differentiation and functions in different manners via transcriptional induction of positive regulators such as Fgf8 and Fgf4, and negative regulators such as Jag2. Therefore, we examined whether TAp63γ negatively regulates limb growth *in vivo*. We generated transgenic mice, *CAG-EGFP-TAp63γ*, which expressed TAp63γ in a Cre recombinase-dependent manner (Fig 7A), and then mated *Msx2-Cre*, *p63*^*fl/fl*^, *p63*^*Δ/+*^, and *CAG-EGFP-TAp63γ* mice. Compared with the phenotype of *Msx2-Cre;p63*^*Δ/fl*^ neonates, the hypoplasia of autopods and distal zeugopods was exacerbated in *Msx2-Cre;p63*^*Δ/fl*^;*CAG-EGFP-TAp63γ* neonates, particularly in hind limbs (Fig 7B and 7C). We then harvested whole limb buds from *p63*^*fl/+*^, *Msx2-Cre;p63*^*Δ/fl*^, and *Msx2-Cre;p63*^*Δ/fl*^;*CAG-EGFP-TAp63γ* littermate embryos at E11.5, and measured mRNA levels of *p63*, *Fgf8*, *Fgf4* and *Jag2*. Expression of *p63* and *Jag2* in the limb buds of *Msx2-Cre;p63*^*Δ/fl*^;*CAG-EGFP-TAp63γ* embryos was significantly increased compared with that in *Msx2-Cre;p63*^*Δ/fl*^ embryos, while expression of *Fgf8* and *Fgf4* was not upregulated (Fig 7D).

## Discussion

The present study showed that ΔNp63 and p63γ were most abundantly expressed in the AER as N- and C-terminal variants, respectively. *Msx2-Cre*-mediated knockout of p63 resulted in limb malformation, which was more obvious in distal elements, accompanied by decreased expression of various AER-related genes. The *in vitro* experiments using *p63*^*fl/fl*^ iPS cells confirmed that expression of Fgf8, Fgf4, and Jag2 was dependent on p63. Promoter analyses and ChIP assays indicated that these genes were direct transcriptional targets of p63. Furthermore, TAp63γ overexpression exacerbated the impairment of limb formation in *Msx2-Cre;p63*^*Δ/fl*^ mice. These present data provide the underlying molecular mechanisms of the striking limb defects in p63-deficient mice shown by previous studies [20, 21], and further revealed different regulation by ΔNp63 and TAp63.

WISH has been a standard method to evaluate gene expression in embryos at early and intermediate stages. In the present study, we employed an *in vivo* cell tracking system and FACS to examine gene expression and alternative splicing in the AER and mesenchyme. Using this combination of the two techniques, we can quantify gene expression and individual mRNA levels of each splicing variant of p63 in a site-specific manner. We sorted cells dissociated from *Prrx1-Cre;Ai14* limb buds, and analyzed mRNA levels of fluorescent-positive and -negative cells by RT-qPCR. The data obtained from this line were validated by appropriate expression of AER or mesenchymal marker genes (Fig 2C). A previous study has shown abundant expression of ΔNp63γ and TAp63γ, and weak expression of ΔNp63α by immunoblotting of whole embryo extracts [21]. We quantified site-specific expression of each p63 transcript variant, and confirmed that the major transcript variants in the AER were ΔNp63 and p63γ (Fig 2D and 2H). Similarly, expression of *Fgf8*, *Fgf4*, *Jag2*, *Dlx5*, *Dlx6*, and *Rspo2* was markedly abundant in AER cells (Fig 2C), which is consistent with the previous expression patterns shown by WISH [4, 7–9, 14]. Furthermore, the difference of *Msx2* mRNA levels in both tissues was relatively small, and expression of *Msx1* in the limb mesenchyme was higher than that in the AER (Fig 2C). These data are compatible with previous results showing that expression areas of Msx1 and Msx2 are broader than the AER [3, 13, 14].

**Fig 8:**
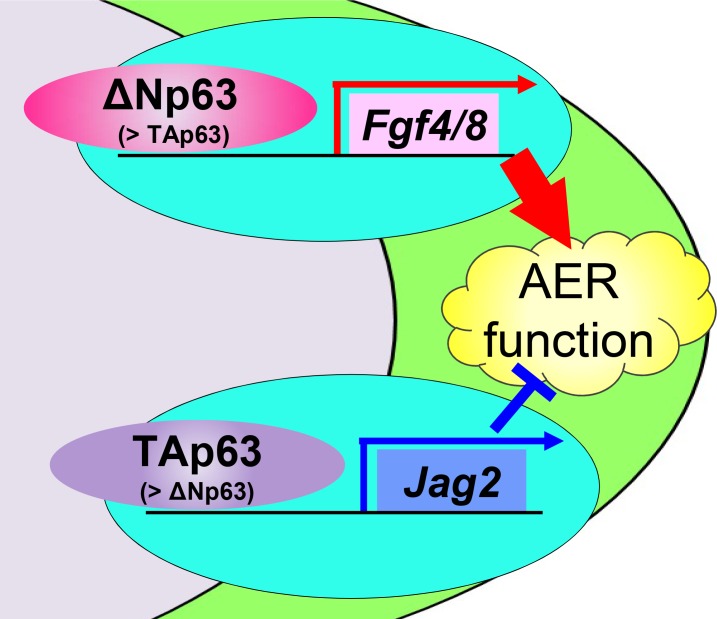
A schematic diagram of different regulation by ΔNp63 and TAp63 in the AER cells.

In the present study, we found that ΔNp63γ and TAp63γ regulate AER functions in different manners. ΔNp63γ regulates Fgf8 and Fgf4, while TAp63γ regulates Jag2 (Fig 8). Although previous studies show that each transcript variant of p63 plays a specific role in various tissues and cell types, different roles performed by different p63 variants in the same tissue had not been reported. The present findings suggest that ΔNp63γ is the most essential variant for growth and maintenance of the AER, because it is the most involved in transcriptional induction of Fgf8 and Fgf4 (Fig 5 and S2 Fig). TAp63γ was increased in the later stages and most involved in Jag2 induction (Figs 1C and 6). Furthermore, AER-specific overexpression of TAp63γ exacerbated the impairment of limb formation by p63 deficiency (Fig 7). These *in vivo* and *in vitro* data indicate that TAp63γ negatively regulates the growth and functions of the AER and may contribute to harmonized limb formation. However, we could not delineate the mechanisms regulating the transcription of each p63 variant in the AER. Elucidation of the upstream molecules or signaling pathways will further our understanding of limb organogenesis and development.

Limb development was markedly impaired in *p63*^*Δ/Δ*^ mice, but normal in *p63*^*Δ/+*^ mice (S1A and S1B Fig). Moreover, *Msx2-Cre;p63*^*Δ/fl*^ neonates displayed malformation of distal limb elements (Fig 3A and 3B). p63 deletion was incomplete and *Fgf8* expression was consequently detected around the tip of the AER because *Msx2* expression might have been weak in this region (Fig 3C). *p63* expression in *Msx2-Cre;p63*^*Δ/fl*^ limb buds remained at about one-third to one-fifth of that in the control (Fig 3D). These data indicate that stylopods and zeugopods can be formed by the *p63* expression level in *Msx2-Cre;p63*^*Δ/fl*^ limb buds, but autopod formation may require more p63.

In conclusion, ΔNp63 and TAp63 control limb development through transcriptional regulation of different essential molecules with different roles in the AER, such as Fgf8, Fgf4 and Jag2. The present methods and findings may contribute to further understanding of the molecular network of limb development.

## Supporting information

S1 Fig p63 knockout mice (*p63*^*Δ/Δ*^) generated by mating *CAG-Cre* mice with *p63*^*fl/fl*^ mice. (A) Gross appearances of WT, heterozygous (*p63*^*Δ/+*^), and homozygous (*p63*^*Δ/Δ*^) mutant E18.5 embryos. Scale bar, 2 mm. Images are representative of *n* = 3 mice per genotype. (B) Double staining with alizarin red and alcian blue of whole skeletons of WT, *p63*^*Δ/+*^, and *p63*^*Δ/Δ*^ mutant E18.5 embryos. Scale bar, 2 mm. Images are representative of *n* = 3 mice per genotype. (C) mRNA levels of *p63* in the whole bodies of WT, *p63*^*Δ/+*^, and *p63*^*Δ/Δ*^ mutant E18.5 embryos. Error bars indicate s.d. (*n* = 3 biological replicates). **P<0.01 (unpaired two-tailed Student's t test).
(PDF)

S2 Fig Transcriptional regulation of Fgf4 by ΔNp63γ. (A) 5′-end flanking region up to −3,468 bp from the TSS of the mouse Fgf4 gene. Four consensus sequences for p63 binding in this region are shown as B1–4. (B) Luciferase activities in B16 melanoma cells co-transfected with a luciferase reporter gene construct containing a fragment (−3,468 bp to 0 bp) of the Fgf4 gene and an expression vector for GFP, ΔNp63γ, or TAp63γ. RLA, relative luciferase activity. Error bars indicate s.d. (*n* = 3 biological replicates). **P<0.01 (unpaired two-tailed Student‘s t test). (C) Luciferase activities in B16 melanoma cells co-transfected with luciferase reporter gene constructs containing the indicated fragments ligated to a miniP and an expression vector for GFP or ΔNp63γ. Error bars indicate s.d. (*n* = 3 biological replicates). *P<0.05, **P<0.01 vs. GFP. ^#^*P*<0.05, ^##^*P*<0.01 vs. WT with ΔNp63γ (unpaired two-tailed Student's t test).
(PDF)

S1 File The ARRIVE Guidelines Checklist. (PDF)

S1 Table List of primers used for genotyping. (PDF)

S2 Table List of primers used for real-time RT-qPCR. (PDF)

S3 Table List of primers used for ChIP-qPCR. (PDF)
