# Supplementary material for: Different regulation of limb development by p63 transcript variants
Source: PLoS One. 2017 Mar 23;12(3):e0174122. doi: 10.1371/journal.pone.0174122 (PMC5363923; doi:10.1371/journal.pone.0174122)
Supplement: S1 Fig — (A) Gross appearances of WT, heterozygous (p63Δ/+), and homozygous (p63Δ/Δ) mutant E18.5 embryos. Scale bar, 2 mm. Images are representative of n = 3 mice per genotype. (B) Double staining with alizarin red and alcian blue of whole skeletons of WT, p63Δ/+, and p63Δ/Δ mutant E18.5 embryos. Scale bar, 2 mm. Images are representative of n = 3 mice per genotype. (C) mRNA levels of p63 in the whole bodies of WT, p63Δ/+, and p63Δ/Δ mutant E18.5 embryos. Error bars indicate s.d. (n = 3 biological replicates). **P<0.01 (unpaired two-tailed Student's t test). (PDF) [file pone.0174122.s001.pdf]

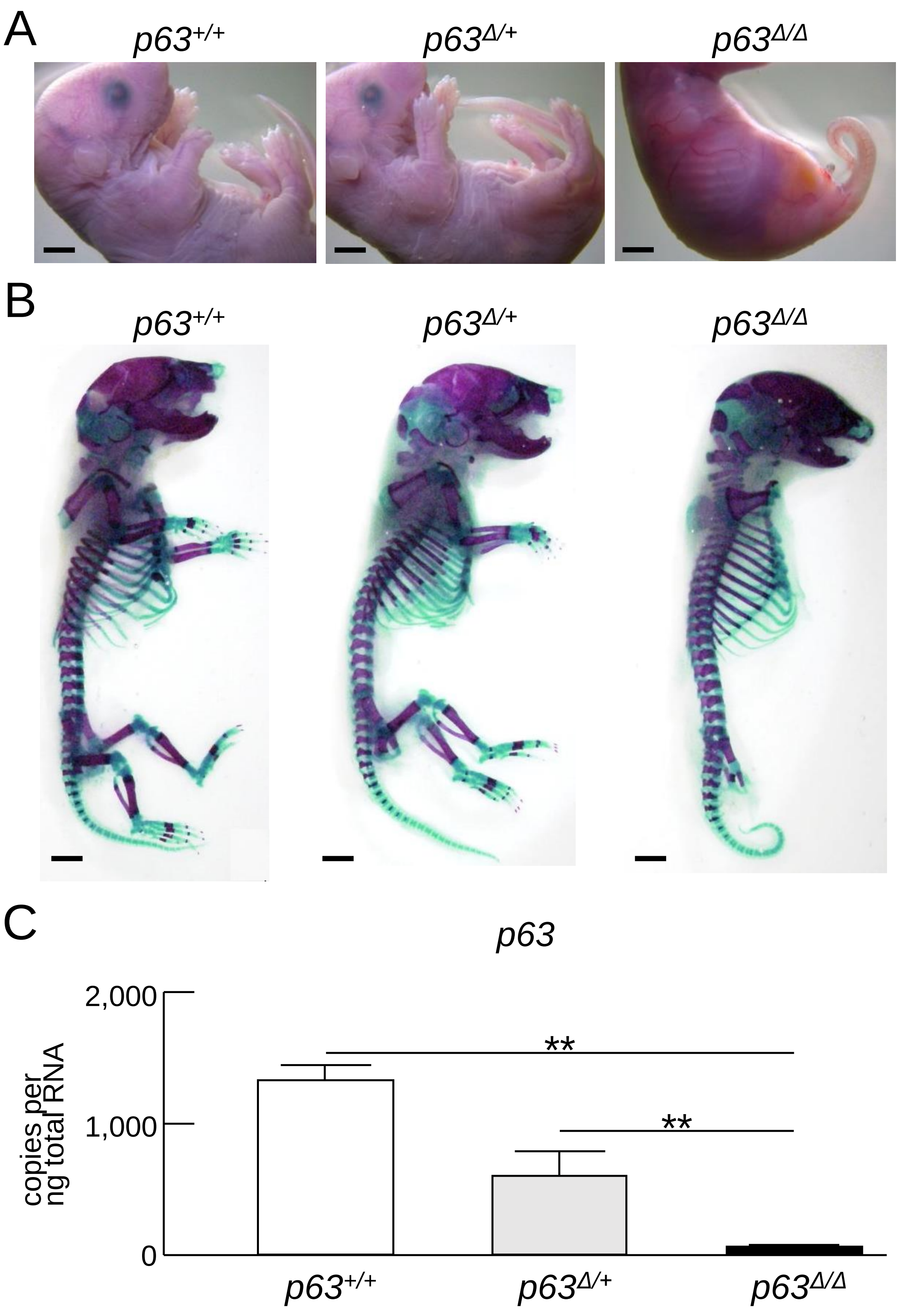

S1 Fig. p63 knockout mice (*$p63^{\Delta/\Delta}$*) generated by mating *CAG-Cre* mice with *$p63^{fl/fl}$* mice. (A) Gross appearances of WT, heterozygous (*$p63^{\Delta/+}$*), and homozygous (*$p63^{\Delta/\Delta}$*) mutant E18.5 embryos. Scale bar, 2 mm. Images are representative of $n = 3$ mice per genotype. (B) Double staining with alizarin red and alcian blue of whole skeletons of WT, *$p63^{\Delta/+}$*, and *$p63^{\Delta/\Delta}$* mutant E18.5 embryos. Scale bar, 2 mm. Images are representative of $n = 3$ mice per genotype. (C) mRNA levels of *p63* in the whole bodies of WT, *$p63^{\Delta/+}$*, and *$p63^{\Delta/\Delta}$* mutant E18.5 embryos. Error bars indicate s.d. ($n = 3$ biological replicates). $^{**}P < 0.01$ (unpaired two-tailed Student's t test).
