# Supplementary material for: Different regulation of limb development by p63 transcript variants
Source: PLoS One. 2017 Mar 23;12(3):e0174122. doi: 10.1371/journal.pone.0174122 (PMC5363923; doi:10.1371/journal.pone.0174122)
Supplement: S2 Fig — (A) 5′-end flanking region up to −3,468 bp from the TSS of the mouse Fgf4 gene. Four consensus sequences for p63 binding in this region are shown as B1–4. (B) Luciferase activities in B16 melanoma cells co-transfected with a luciferase reporter gene construct containing a fragment (−3,468 bp to 0 bp) of the Fgf4 gene and an expression vector for GFP, ΔNp63γ, or TAp63γ. RLA, relative luciferase activity. Error bars indicate s.d. (n = 3 biological replicates). **P<0.01 (unpaired two-tailed Student‘s t test). (C) Luciferase activities in B16 melanoma cells co-transfected with luciferase reporter gene constructs containing the indicated fragments ligated to a miniP and an expression vector for GFP or ΔNp63γ. Error bars indicate s.d. (n = 3 biological replicates). *P<0.05, **P<0.01 vs. GFP. #P<0.05, ##P<0.01 vs. WT with ΔNp63γ (unpaired two-tailed Student's t test). (PDF) [file pone.0174122.s002.pdf]

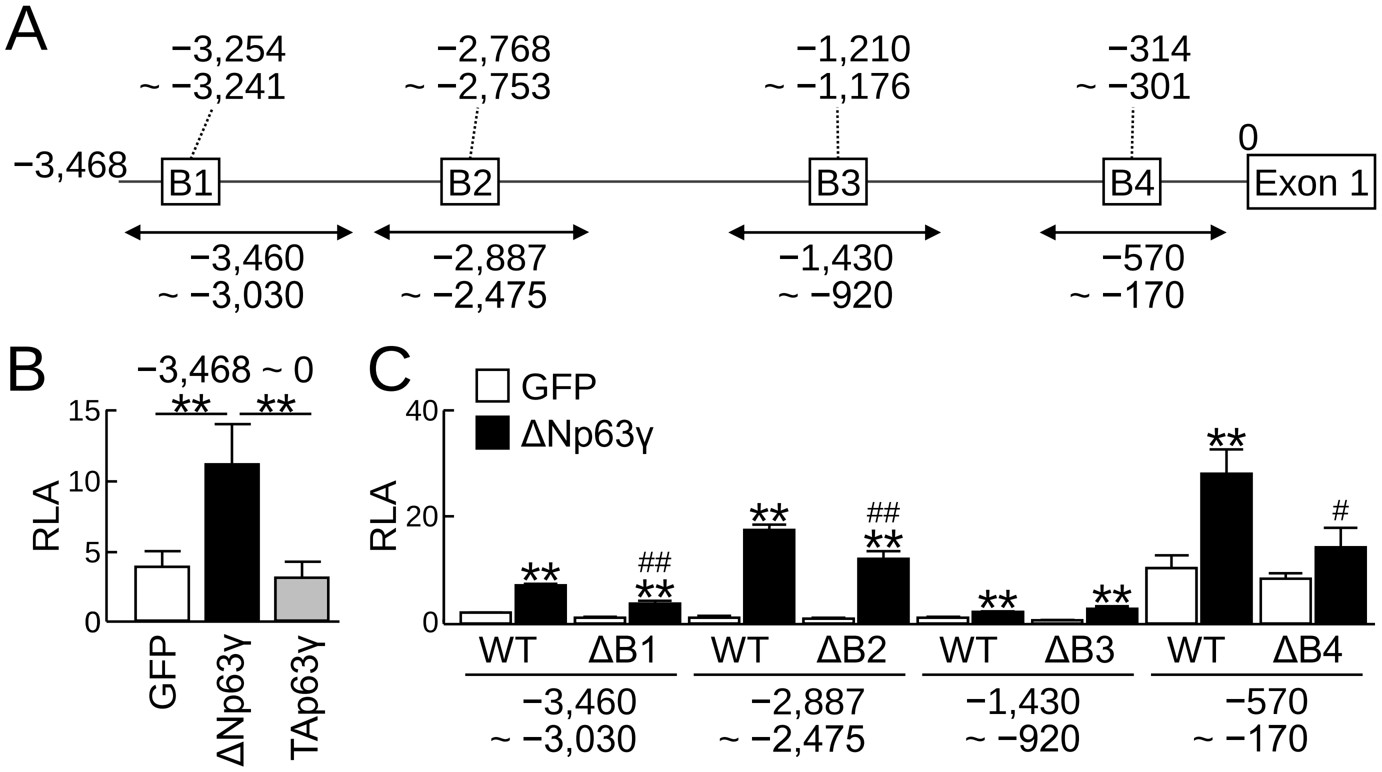

S2 Fig. Transcriptional regulation of Fgf4 by ΔNp63γ. (A) 5′-end flanking region up to −3,468 bp from the TSS of the mouse Fgf4 gene. Four consensus sequences for p63 binding in this region are shown as B1–4. (B) Luciferase activities in B16 melanoma cells co-transfected with a luciferase reporter gene construct containing a fragment (−3,468 bp to 0 bp) of the Fgf4 gene and an expression vector for GFP, ΔNp63γ, or TAp63γ. RLA, relative luciferase activity. Error bars indicate s.d. (*n* = 3 biological replicates). ***P* < 0.01 (unpaired two-tailed Student‘s t test). (C) Luciferase activities in B16 melanoma cells co-transfected with luciferase reporter gene constructs containing the indicated fragments ligated to a miniP and an expression vector for GFP or ΔNp63γ. Error bars indicate s.d. (*n* = 3 biological replicates). **P* < 0.05, ***P* < 0.01 vs. GFP. $^{\#}P < 0.05$, $^{\#\#}P < 0.01$ vs. WT with ΔNp63γ (unpaired two-tailed Student's t test).
