# Supplementary material for: Different regulation of limb development by p63 transcript variants
Source: PLoS One. 2017 Mar 23;12(3):e0174122. doi: 10.1371/journal.pone.0174122 (PMC5363923; doi:10.1371/journal.pone.0174122)
Supplement: S1 Table — (PDF) [file pone.0174122.s004.pdf]

S1 Table. List of primers used for genotyping.

Allele	Primer sequences (forward and reverse, 5'-3')
p63-flox	AAGTGGCAGTGAGCAGAAC
	ACAATTCCAGTCAAACATCAA
p63-delta	TTACTAGGTGCCCACTCTTG
	ACAATTCCAGTCAAACATCAA
Ai14-mutant	GGCATTAAAGCAGCGTATCC
	CTGTTCCTGTACGGCATGG
Ai14-wlidtype	AAGGGAGCTGCAGTGGAGTA
	CCGAAAATCTGTGGGAAGTC
Prrx1-Cre	CTCCCTCCTCCTCTCTTGCT
	AGGCAAATTTTGGTGTACGG
Msx2-Cre	CCATGCCCTCCGCAGATTTCC
	GTTATTCAACTTGCACCATGC
CAG-Cre	CCTACAGCTCCTGGGCAACGTGC
	CTAATCGCCATCTTCCAGCAGG
CAG-EGFP	ACGTAAACGGCCACAAGTTC
	GTCCTCCTTGAAGTCGATGC
