# Supplementary material for: Different regulation of limb development by p63 transcript variants
Source: PLoS One. 2017 Mar 23;12(3):e0174122. doi: 10.1371/journal.pone.0174122 (PMC5363923; doi:10.1371/journal.pone.0174122)
Supplement: S2 Table — (PDF) [file pone.0174122.s005.pdf]

S2 Table. List of primers used for real-time RT-qPCR.

Gene	Primer sequences (forward and reverse, 5'-3')
β-Actin	AGATGTGGATCAGCAAGCAG
	GCGCAAGTTAGGTTTTGTCA
p63	TGAGCCGTGAGTTCAATGAG
	ACCTGTGGTGGCTCATAAGG
ΔNp63	CTGGAAAACAATGCCCAGAC
	GAGGAGCCGTTCTGAATCTG
TAp63	CCCAGAGGTCTTCCAGCATA
	TTTTCGGAAGGTTCATCCAC
p63α	ATCTCCTGAGGACCCCAAGT
	CTTTGATACGCTGCTGCTTG
p63β	GCATTGTCAGGATTTGGCAAG
	GTCTCACTGGAGCCCACACT
p63γ	GCACACGATCGAAACGTACA
	ATTCCTGAAGCAGGCTGAAA
Fgf8	TTGGAAGCAGAGTCCGAGTT
	TGTGAATACGCAGTCCTTGC
Fgf4	GGGTTTGATGTTTCTGCTCTG
	CAGTCCATGGGGCTGTCTAT
Msx1	CTCTCGGCCATTTCTCAGTC
	TACTGCTTCTGGCGGAACTT
Msx2	AACACAAGACCAACCGGAAG
	GCAGCCATTTTCAGCTTTTC
Jag2	CAGATCCGAGTACGCTGTG
	GGCTTCTTTGCATTCTTTGC
Dlx5	CTGGCCGCTTTACAGAGAAG
	CTGGTGACTGTGGCGAGTTA
Dlx6	ACCATCGCTTTCAGCAGACT
	AGAAACGTCCCACACTGGAG
Rspo2	ACCGATGGAGACGCAATAAG
	TGCAGGCACTCTCCATACTG
Prrx1	GCGGAGAAACAGGACAACAT
	ACTTGGCTCTTCGGTTCTGA
