# Supplementary material for: Different regulation of limb development by p63 transcript variants
Source: PLoS One. 2017 Mar 23;12(3):e0174122. doi: 10.1371/journal.pone.0174122 (PMC5363923; doi:10.1371/journal.pone.0174122)
Supplement: S3 Table — (PDF) [file pone.0174122.s006.pdf]

S3 Table. List of primers used for ChIP-qPCR.

Gene	Region	Primer sequences (forward and reverse, 5'-3')
Fgf8	P1	CATTAGCAGAGGATCGAATGG
		TGTACGACCACGAGCAGTTT
	P2	ATTTCTGGCCTGTTTTCCTG
		AGAAGGCAAATGTGGATTGG
	P3	GTGTTTGCAGGAGGGAGTGT
		ACAGCCTGGCCACCTTTC
	P4	TGCAAGATGGGATGCATAAA
		AGGCTAAAGAGAGGCAAGCA
	P5	CTCCCTCCCAGAGCCTCTTT
		CGGTGCCTTCACCTTTCTAA
	P6	GGAAGAGACAGGCAAGGGTA
		CAGGGGAGGGGTCTAGGG
	P7	CCTCCCTCTTCCAAGTGACA
		CCTTTCCCCTTCTGAGTTCC
	P8	GCCCTTTGCATTCTCAACAT
		AGAGGAGCAGGGTGCTTTTA
Jag2	Q1	TGCGCTGCCTTATTTTTAGG
		CTCCCCGTTCACGTTCC
	Q2	CGCGTGTGCCTTAAGGAGT
		CGCCGTAGCCGTAGCTG
	Q3	CCCTGCAGTTTCCTGGATG
		TCTGCCAACACACACAATCA
	Q4	GCCCTAGGCAGTTACTATTGG
		GCCCAAGTGCAGATGACAG
	Q5	CTGCAGGAAGGAGCATTTGT
		CGCAGCCTCCTGTTACTTTC
	Q6	GGCGAGCGAGCTGTCAGT
		GTCCTGGCCTCGGCTCT
	Q7	TGCTTGATCTGCCACAAGTT
		GTTCTTGCTGTCCCCAGGTA
Negative control primers		TCCTGGGGGTCTACAACAAG
		CCACCCCTCCAAATCTTACA
